# Case Report: Rhabdomyolysis secondary to vildagliptin overdose in a suicidal attempt: A case report and brief literature review

**DOI:** 10.3389/fphar.2022.955162

**Published:** 2022-08-10

**Authors:** Zhijia Tang, Xiaofang Fan, Zhen Feng, Bing Han, Nan Guo

**Affiliations:** ^1^ Minhang Hospital & Department of Clinical Pharmacy, School of Pharmacy, Fudan University, Shanghai, China; ^2^ Department of Endocrinology, Minhang Hospital, Shanghai, China

**Keywords:** vildagliptin, rhabdomyolysis, DPP-4 inhibitor, overdose, case report

## Abstract

Rhabdomyolysis is a life-threatening syndrome associated with direct or indirect muscle damage that is rarely reported with dipeptidyl peptidase (DPP)-4 inhibitors. Here we presented a case in which a 58-year-old female suffered from severe swelling and pain in bilateral lower limbs and oliguria after a suicidal vildagliptin overdose. Drug-induced rhabdomyolysis and drug-induced liver injury were diagnosed based on laboratory and radiological findings. The patient was treated with fluid resuscitation, insulin, electrolyte replacement, diuretics, urine alkalizing agents, anticoagulants, antioxidants, and 24-h bedside ECG monitoring and suicide prevention. After 20 days of hospitalization and close monitoring, the patient was discharged without sequelae. Risk factors, diagnostic criteria, disease mechanisms, and outcomes were also discussed. This case illustrated that overdose of oral anti-diabetic medications may result in clinically significant adverse events, such as rhabdomyolysis in this case with a DPP-4 inhibitor. Although the incidence is low, special attention should be paid to intentional or accidental exposure to anti-diabetic medications during suicide attempts, especially in depressed patients with diabetes.

## Introduction

Rhabdomyolysis is a serious syndrome in which muscle contents (such as myoglobin) leak into the bloodstream due to the breakdown of skeletal muscle fibers (Sauret et al., 2002). An elevated level of creatine kinase (CK) (i.e., >1,000 IU/L) is the most sensitive laboratory test to assess the severity of muscle damage that causes rhabdomyolysis (Huerta-Alardin et al., 2005; Cervellin et al., 2010). The typical symptoms of rhabdomyolysis are muscle pain, weakness, swelling, nausea, vomiting, confusion, dark urine, and oliguria. Approximately 10–40% of patients with rhabdomyolysis will develop further kidney damage, such as acute kidney injury (AKI), which may require dialysis (Kasaoka et al., 2010). Although trauma and muscle compression remain the two most common causes of direct muscle injury, drug-induced rhabdomyolysis may also result from certain classes of drugs. Previous studies have shown that the drugs most likely to cause rhabdomyolysis include psychiatric agents, antihistamines, illicit substances, and those affecting the cytochrome P450 system, such as statins (Sauret et al., 2002; Hohenegger, 2012). In addition, concomitant use of Chinese herbal medicine and herbal dietary supplements (HDS) has been shown to be associated with rhabdomyolysis based on case reports and spontaneous reports of suspected adverse events (Li et al., 2020; Allkanjari et al., 2022). Dipeptidyl peptidase (DPP)-4 inhibitors, one of the safest oral hypoglycemic agents that reduce the degradation of GLP-1, are usually not considered to cause rhabdomyolysis (Mulvihill and Drucker, 2014; Marx et al., 2021). Other risk factors of rhabdomyolysis include old age, female, strenuous exercise, frailty, kidney or liver insufficiency, and diabetes (Sauret et al., 2002; Huerta-Alardin et al., 2005).

Here, we report a clinical case of rhabdomyolysis caused by vildagliptin overdose in a depressed patient with type 2 diabetes mellitus (T2DM) trying to commit suicide, and how diagnosis and treatment were performed. This adverse event has been reported to the National Adverse Drug Reaction Monitoring System (www.adrs.org.cn) under the affiliation of China’s National Medical Products Administration (notification number: 310112-1-027962-2022-00059).

### Case presentation

**FIGURE 1:**
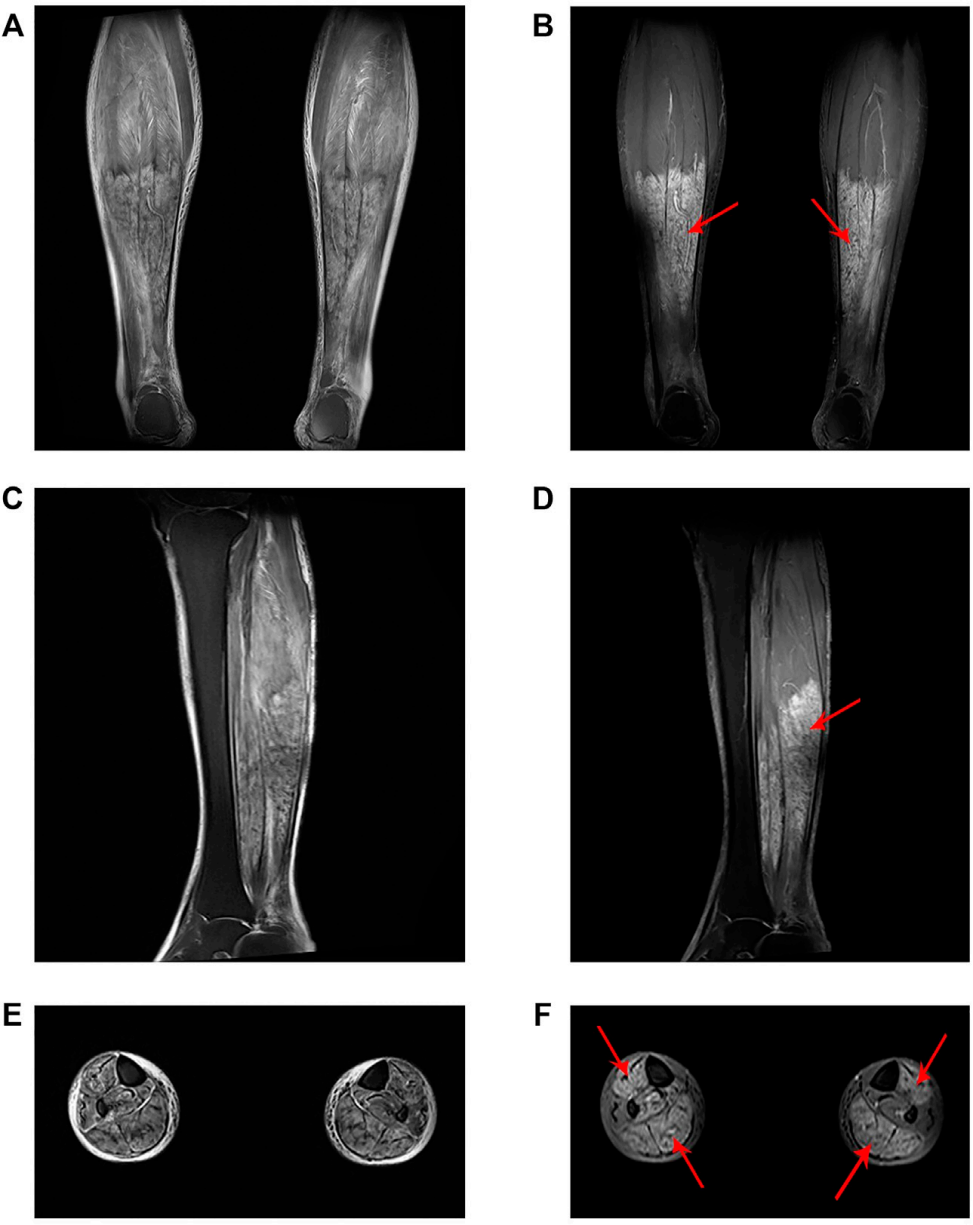
Lower limb muscle MRI findings. **(A)** Coronal, **(C)** sagittal and **(E)** axial T2-weighted images. **(B)** Coronal, **(D)** sagittal and **(F)** axial T1-weighted fat-suppressed contrast-enhanced images demonstrated high signal intensity of the tibialis anterior, gastrocnemius and soleus with homogenous enhancement (red arrow).

A 58-year-old female presented to our emergency department in a wheelchair with severe pitting edema and pain in both lower extremities as well as oliguria after a suicidal attempt that she took vildagliptin 1,400 mg (14-times the normal dosage). Her height and weight were 156 cm and 50 kg, respectively; temperature, 37.1°C; blood pressure, 120/60 mm Hg; pulse, 80 beats per minute; respiratory rate, 19 breaths per minute. She had a known history of T2DM, hypertension, and depression. Home medications included vildagliptin, insulin aspart and nifedipine. No psychiatric medication or psychotherapy was received. The patient recalled no heavy exercise, muscle injuries, toxins, alcohol abuse, or traumatic accident recently. Neither previous suicide attempts nor family history of suicide was noticed. Physical examination revealed diminished Dorsalis pedis pulse and sensation in bilateral feet. The immunology tests showed substantially increased white blood cell (31.34 × 10^9^/L), neutrophil percentage (92.5%), procalcitonin (1.921 ng/ml) and C-reactive protein (159.69 mg/L). The biochemical analyses disclosed a high level of creatine kinase (CK) (11,311 U/L), myoglobin (>500 ng/ml) and blood urea nitrogen (BUN) (8 mmol/L). Considering the increase in CK (>87 times the upper limit of normal [ULN]) and myoglobin (>7 × ULN), and the very typical clinical manifestations, a preliminary diagnosis of rhabdomyolysis was made. The magnetic resonance imaging (MRI) findings also revealed abnormal signal intensity of muscles in the anterior and posterior compartments bilaterally (Figure 1).

**FIGURE 2:**
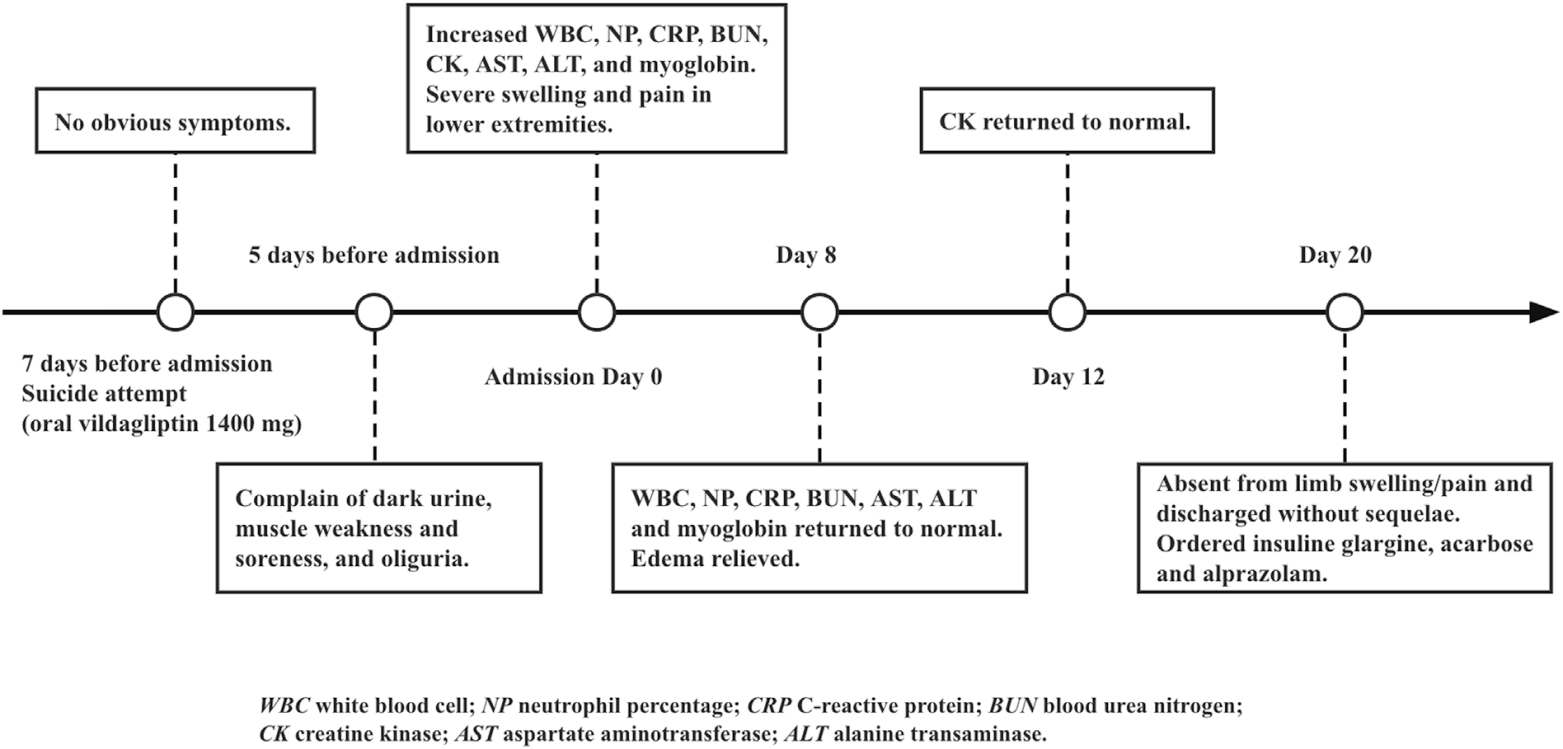
Timeline of the case report.

Lactic acidosis was assumed on elevated blood lactate (6.24 mmol/L), low base excess (-5.0 mmol/L) and low pH value (7.231). The liver function test reported an elevated aspartate aminotransferase (375 U/L) and alanine transaminase (214 U/L). Diabetic ketoacidosis (DKA) was diagnosed based on increased lactate dehydrogenase (1,249 U/L), blood ketone (4.4 mmol/L), and random blood glucose (25.5 mmol/L). In the emergency room, the patient received aggressive fluid resuscitation of normal saline and furosemide to eliminate myoglobin from the body, intravenous infusion of regular insulin and potassium chloride to control DKA, sodium bicarbonate injection to correct acidemia and achieve urinary alkalinization, and cefminox injection to treat *Streptococcal viridans*, *Neisseria sicca* and *E. coli* infection. On top of that, she also received intravenous α-lipoic acid and glutathione to reduce oxidative stress, and subcutaneous dalteparin to prevent blood clots due to an elevated D-dimer level (1,780 ng/ml), as well as 24-h bedside ECG monitoring and suicide prevention. The patient did not show signs of renal failure or cardiac arrhythmia throughout the course. After urinary output boosted to 3,300 ml per day with unnoticeable myoglobin and ketone, she was transferred to the inpatient unit for further observation (Table 1). The timeline of the case was illustrated in Figure 2. The patient was compliant and expressed understanding with all treatment received.

**TABLE 1:** Laboratory test results during patient hospitalization.

	Hospital day		
	Day 0	Day 8	Day 20
Sodium, mmol/L	137	142	141
Potassium, mmol/L	4.70	4.40	4.34
WBC, ×10^9^/L	31.34	6.68	6.62
NP, %	92.5	70.5	66.7
CRP, mg/L	159.69	5.90	1.17
Procalcitonin, ng/mL	1.92		0.04
BUN, mmol/L	8.00	5.10	5.24
AST, U/L	375	34	14
ALT, U/L	214	59	10
CK, U/L	11,311	526	68
Myoglobin, ng/mL	>500	47.86	
LDH, U/L	1,249	272	168
Glucose, mmol/L	25.5 (random)	9.0 (fasting)	6.4 (fasting)
Blood ketone, mmol/L	4.4	<0.6	<0.6

WBC, white blood cell; NP, neutrophil percentage; CRP C-reactive protein; BUN, blood urea nitrogen; AST, aspartate aminotransferase; ALT, alanine transaminase; CK, creatine kinase; LDH, lactate dehydrogenase.

## Discussion

Rhabdomyolysis is a serious syndrome associated with muscle injury. Generally speaking, the mortality rate is low, but it may still cause lifelong kidney damage or even death if not treated in time. This patient had extremely high levels of CK and serum myoglobin (>87 times ULN; >7 × ULN, respectively), with very typical clinical manifestations (limb swelling, pain and diminished pedal pulses) and abnormal diagnostic radiographic results (Figure 1). Therefore, although it is currently reported in the literature that 50% of patients with rhabdomyolysis will not complain of muscle pain or weakness, and less than 10% of patients will have typical symptoms (Torres et al., 2015), the diagnosis in this case was clear.

Vildagliptin is a DPP-4 inhibitor, which is rare to induce rhabdomyolysis compared with traumatic causes (Mathieu and Bollaerts, 2007; Strain et al., 2013; Mulvihill and Drucker, 2014; Mathieu et al., 2017). In 2014 and 2017, the European Medicines Agency (EMA) and US Food and Drug Administration (FDA) raised warnings about the possible rhabdomyolysis side effect for this class of drugs (European Medicines Agency Pharmacovigilance Risk Assessment Committee, 2014; Food and Drug Administration, 2017). However, there was no consensus on the risk and underlying mechanism of DPP-4 inhibitors because most reported cases were related to concurrent use of high-risk drugs such as statins, Chinese herbal medicines, and dietary supplements which were known to cause rhabdomyolysis (Kao et al., 2008; DiGregorio and Pasikhova, 2009; Bhome and Penn, 2012; Labat et al., 2017). Notably, this patient never took any known high-risk agents concomitantly. According to EMA Summary of Product Characteristics (SmPC), there is very limited information on whether an overdose of vildagliptin causes rhabdomyolysis and how to treat it (European Medicines Agency, 2022). In a rising dose tolerability study, a healthy subject who was given vildagliptin 600 mg (6 times the normal dose) for 10 days experienced feet and hand edema, as well as increased levels of CK, AST, CRP and myoglobin (European Medicines Agency, 2022). The subject recovered without any treatment after discontinuation of the drug. Other studies have also confirmed that musculoskeletal disorders caused by DPP-4 inhibitors are usually mild and can be relieved on their own after stopping the drug (Tarapues et al., 2013). The underlying mechanism may be explained by the wide distribution of DPP-4 in striated muscle and the lower pain threshold in rhabdomyolysis (Guieu et al., 2006; Tarapues et al., 2013). Despite the lack of direct references, considering that the patient had been taking vildagliptin for many years without any complaints, we can speculate on the causal relationship between the overdose and the occurrence of rhabdomyolysis in this case.

Generally, intravenous fluid therapy is the cornerstone of treatment. In our case, because the patient’s suicide attempt happened a few days ago, we did not perform gastric lavage or hemodialysis. There is no known antidote, and supportive management is recommended in the case of overdose (European Medicines Agency, 2022). An intravenous bolus furosemide was used along with normal saline to help maintain urine production and thus prevent kidney failure. The use of sodium bicarbonate is controversial based on literature. However, since vigorous fluid repletion with non-buffered crystalloids may contribute to acidosis, and our patient already had an episode before, urinary alkalinization with close monitoring of acid-base status and electrolytes (especially potassium) should be a reasonable approach. Furthermore, bicarbonate has demonstrated possible benefits in reversal of mild to moderate acidemia (Chua et al., 2011), which may be another reason to support its use in our situation. The patient’s DKA was assumed to be triggered by inadequate glycemic control and infection, and was resolved with fluid resuscitation and insulin and potassium replacement. In addition, some studies have declared that antioxidants and free radical scavengers could not only reduce oxidative damage in liver injury, but also decrease the risk of myoglobinuric acute kidney injury in rhabdomyolysis (Bosch et al., 2009). Therefore, we administered α-lipoic acid and glutathione until the patient’s liver function recovered.

In this case, the patient had been suffering from depression for years without receiving psychiatric medications or psychotherapy. Previous studies have showed that people with chronic diseases such as T2DM often experience depression and anxiety, which can lead to suicidal behavior (Semenkovich et al., 2015; Kim et al., 2022). Risky drugs include high doses of insulin, metformin, glipizide, liraglutide and sitagliptin (Sarkar and Balhara, 2014; Goonoo et al., 2020; Tuncali et al., 2021). Our case further indicated that vildagliptin is also a potential drug for suicide and self-harm, which should be particularly noted in depressed individuals. Although after consultation with a psychiatrist, we considered this patient to be at low risk for another suicide, we implemented 24-h monitoring and suicide prevention during hospitalization. Additionally, given her inadequate glycemic control, we added a long-acting insulin (insuline glargine) to the patient’s home medication, along with acarbose and the antidepressant alprazolam. Vildagliptin was discontinued at discharge.

## Conclusion

In conclusion, we reported a rare case of vildagliptin overdose-induced rhabdomyolysis accompanied by DKA, infection and liver damage, and provided some hints on how to treat such condition. This case showed that overdose of oral anti-diabetic medications, including DPP-4 inhibitors, may result in severe adverse events, such as rhabdomyolysis, and such intentional or accidental drug exposure should be especially noted in depressed patients during suicide attempts.

## Data Availability

The original contributions presented in the study are included in the article/Supplementary Material, further inquiries can be directed to the corresponding authors.
